# Posterior unilateral small fenestration of lamina combined with a custom-made Y-shaped fracture reduction device for the treatment of severe thoracolumbar burst fracture: a prospective comparative study

**DOI:** 10.1186/s13018-023-03971-7

**Published:** 2023-07-25

**Authors:** Zheng Zeng, Dan Zhang, Fen-Lian Zeng, Jun Ao

**Affiliations:** 1 Department of Orthopaedic Surgery, The Second Affiliated Hospital of Zunyi Medical University, Zunyi, 563000 Guizhou China; 2 Department of Orthopaedic Surgery, Affiliated Hospital of Zunyi Medical University, Zunyi, 563000 Guizhou China; 3 Department of Nursing, Affiliated Hospital of Zunyi Medical University, Zunyi, 563000 Guizhou China

**Keywords:** Thoracolumbar burst fracture, Reductor, Surgery

## Abstract

**Background:**

The purpose was to evaluate the clinical effect of a custom-made Y-shaped fracture fragment reduction device and to assist in posterior unilateral small fenestration of lamina to reduce the fracture fragments.

**Methods:**

In this study, 40 patients were assigned to one of two groups: the traditional reduction device group (TRG) or the Y-shaped reduction device group (YRG). All patients underwent posterior unilateral small fenestration of the lamina and direct decompression through the spinal canal. And the operation time (OT), intraoperative bleeding (IB), preoperative, postoperative, and final follow-up data on the spinal stenosis rate (SSR), Cobb angle, the anterior compression ratio of injured vertebrae (ACRIV), and ASIA neurological function grade were compared between the two groups.

**Result:**

There were no complications, including vascular and nerve injury, serious postoperative infection, internal fixation fracture, or loosening, for any of the patients. And the average follow-up time of the two groups was 14.2 months, the average operation time of the TRG was 236.6 min, and the average intraoperative blood loss was 357.20 ml. Moreover, the average operation time of the YRG was 190.6 min, and the average intraoperative blood loss was 241.5 ml. There were significant differences between the two groups in terms of operation duration and intraoperative blood loss. The YRG's was lower than that of the TRG. Besides, there was no difference in SSR, Cobb angle, ACRIV, or neurological recovery between the two groups before or immediately after the operation or at the last follow-up.

**Conclusion:**

The Y-shaped fracture reduction device can reduce the fracture fragments and the OT and IB stably; it also has satisfactory postoperative curative effects and clinical utility.

## Introduction

Thoracolumbar fractures are the most prevalent type of spinal injury, accounting for approximately 50–90% of spinal fractures [1, 2]. Among them, burst fractures attribute to approximately 10–20% [3–5]. 50–60% of patients with thoracolumbar burst fracture will suffer nerve and vascular damage due to the fracture block protruding into the spinal canal, leading to variable nerve dysfunction [6–8]. Moreover, surgical intervention is generally recommended for the treatment of unstable burst fractures, particularly in patients with neurological dysfunction [9, 10].

There are numerous surgical procedures for the treatment of thoracolumbar burst fractures in clinics, yet the posterior approach has become the mainstream surgical method due to its advantages of safety, less trauma, as well as short operation time [11, 12]. Notwithstanding, the traditional posterior approach to the reduction of intraspinal fracture fragments requires complete laminectomy, exposing the spinal canal for reduction, which will cause severe damage to the skeletal structure and soft tissues. Furthermore, unilateral small fenestration of vertebral lamina can reduce the fracture fragments based on destroying the posterior column of the vertebral body and retaining the stability of the vertebral body. Hence, it is utilized frequently in clinical practice.

Studies have demonstrated that [13, 14] when the stenosis rate of the spinal canal is over 50%, not only is the posterior longitudinal ligament damaged, but the fracture block protruding into the spinal canal is likewise overturned. With pedicle screws alone, it is hard to eliminate the fracture fragments in this type of fracture [15, 16]. Moreover, it is frequently necessary to resect the vertebral lamina and use instruments to directly reduce the fracture fragments. Nonetheless, there is no specially designed reduction to assist the unilateral small fenestration of vertebral lamina in the reduction of fracture fragments. Certain reducers need to be reset based on total or semi-laminectomy, which will damage the stability of the spine and increase surgical trauma [17, 18]. For other reducers, despite the fact that they can be reset through small fenestration of lamina, the reset process is not stable, which increases the risk of damaging the dural sac or nerve root [19]. Consequently, it has tremendous clinical significance to design an instrument that assists to reduce the fracture fragments safely and stably via minimally invasive surgery.

**Fig. 1:**
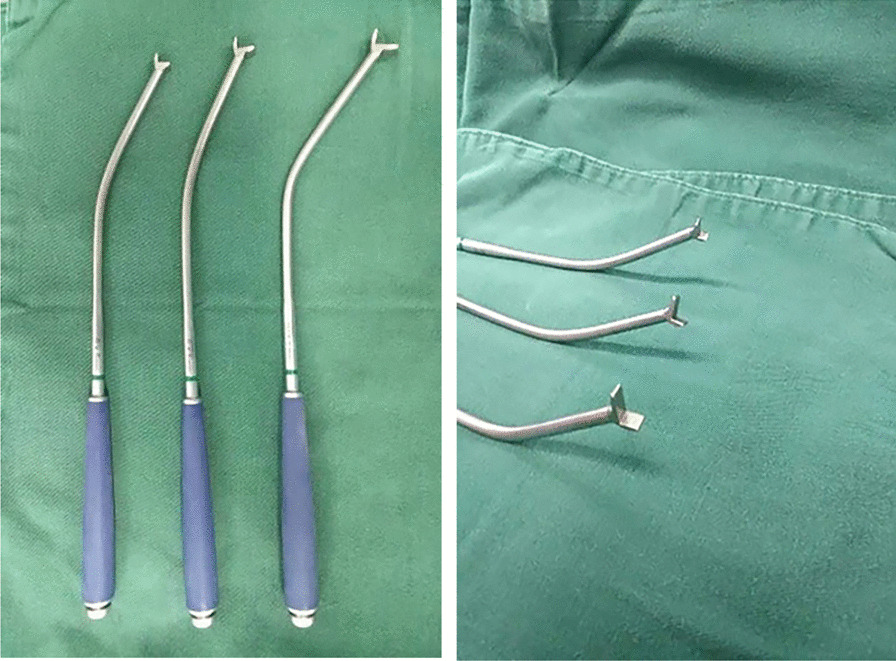
Y-shaped fracture block reduction device

In this study, we designed a Y-shaped fracture fragment reduction instrument based on the morphological characteristics of the fracture fragment (Fig. 1) and evaluated the Y-shaped fracture fragment reduction device used herein in unilateral posterior small laminectomy compared with the traditional reduction device to elaborate its reduction effect.

## Materials and methods

### Patients

From January 2016 to December 2019, every patient underwent surgical treatment in our hospital. And the inclusion criteria were as follows: (1) single segment thoracolumbar burst fracture, with a TLICS score larger than 4 points; (2) rate of spinal canal stenosis caused by the protrusion of vertebral fracture mass into the spinal canal ≥ 50%; (3) the shape of the fracture block protruding into the spinal canal is triangular with turnover; (4) there were no obvious serious injuries to the vertebral bodies adjacent to the injured vertebral body; and (5) the nucleus pulposus of intervertebral disk did not protrude into the spinal canal. The exclusion criteria were (1) patients with pathological fractures, (2) patients with multilevel thoracolumbar fractures, (3) patients with thoracolumbar fracture and dislocation, and (4) patients with severe systemic diseases and physical conditions that prevented them from undergoing surgery, in addition to patients with other surgical contraindications. Forty patients were included; there were 15 males and 5 females with an average age of 49.45 ± 14 years in the YRG, 5 cases involving T_12_, 6 cases involving L_1_, 6 cases involving L_2_, 2 cases involving L_3_, and 1 case involving L_4_. Moreover, there were 2 cases of falling injury, 2 of heavy injury, 2 of car accident injuries, 14 of high falling injuries, and 11 of multiple injuries. According to the ASIA classification, there were 4 cases of grade A, 2 cases of grade B, 5 cases of grade C, 8 cases of grade D and 1 case of grade E. Besides, there were 13 males and 7 females with an average age of 43.1 ± 7.6 years in the TRG, 3 cases involving T_12_, 10 cases involving L_1_, 4 cases involving L_2_, 2 cases involving L_3_, and 1 case involving L_4_. Subsequently, there were 2 cases of a fall injury, 5 cases of traffic accident injury, 13 cases of a high fall injury, and 10 cases of multiple injuries. Neurological function was graded as follows: grade A in 3 cases, grade B in 2 cases, grade C in 7 cases, grade D in 6 cases, and grade E in 2 cases. Table 1 displays the general information of the two groups.

**Table 1:** Comparison of the general preoperative data between the two groups

Groups	Fracture segment					AVRIV (%)	Cobb angle (°)	SSR (%)
	T_12_	L_1_	L_2_	L_3_	L_4_			
YRG	5	6	6	2	1	58.9 ± 12.9	16.11 ± 7.1	58.5 ± 10.4
TRG	4	2	5	8	1	54.7 ± 11.5	15.55 ± 6.8	54.8 ± 7.8
*P*	0.766					0.296	0.800	0.213

### Application principle of the Y-shaped fracture block reduction device

**Fig. 2:**
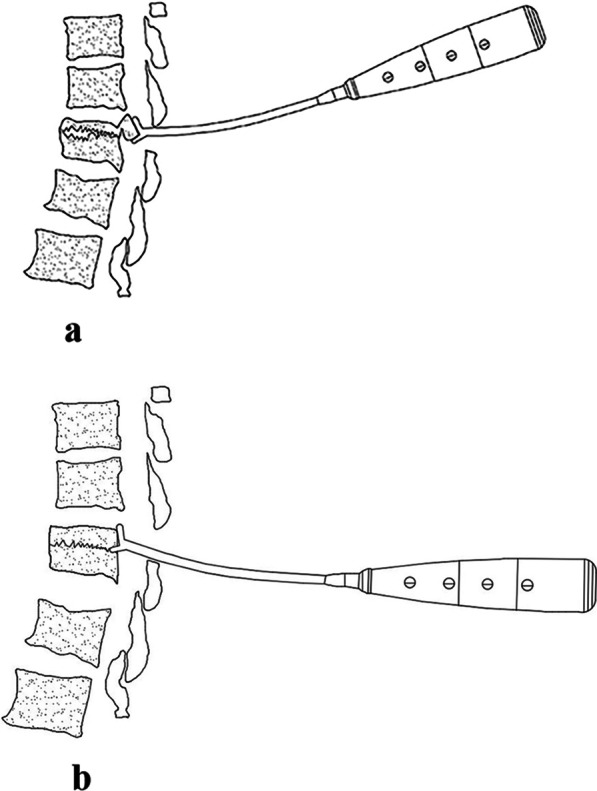
**a** Schematic diagram of the Y-shaped fracture block before reduction. **b** Schematic diagram of the Y-shaped fracture block after reduction

According to the preoperative CT findings of patients with thoracolumbar burst fracture, the prominent portion of the fracture block was determined, and then in preparation for unilateral small laminectomy, the upper edge of the injured vertebral lamina and the lower edge of the upper vertebral lamina were partially resected. After the dural sac and nerve root were protected, the upper and lower parts of the “Y” type anastomotic stoma of the reductor were anastomosed with the cortical surface and cross section of the fracture block through the spinal canal through the window. In addition, the proximal end of the reduction device was aligned perpendicular to the tangent of the injured vertebrae. After stabilization, the rod part was pushed by the handle. It pushed the long piece and the short piece to make the fracture piece in contact with the long piece. The short piece and long and short films were used to reduce the fracture block by pressing it into the vertebral body. At this time, the distal end of the reduction was perpendicular to the tangent line of the injured vertebral body (Fig. 2a, b). All reducers were designed in accordance with the anatomical structure of the spine and the shape characteristics of the fracture block. The thickness of the upper and lower blades at the front end of the reducers was 3 mm, and the opening height was less than 5 cm. When combined with small lamina space fenestration, it could safely reduce the fracture block.

### Surgical procedures

**Fig. 3:**
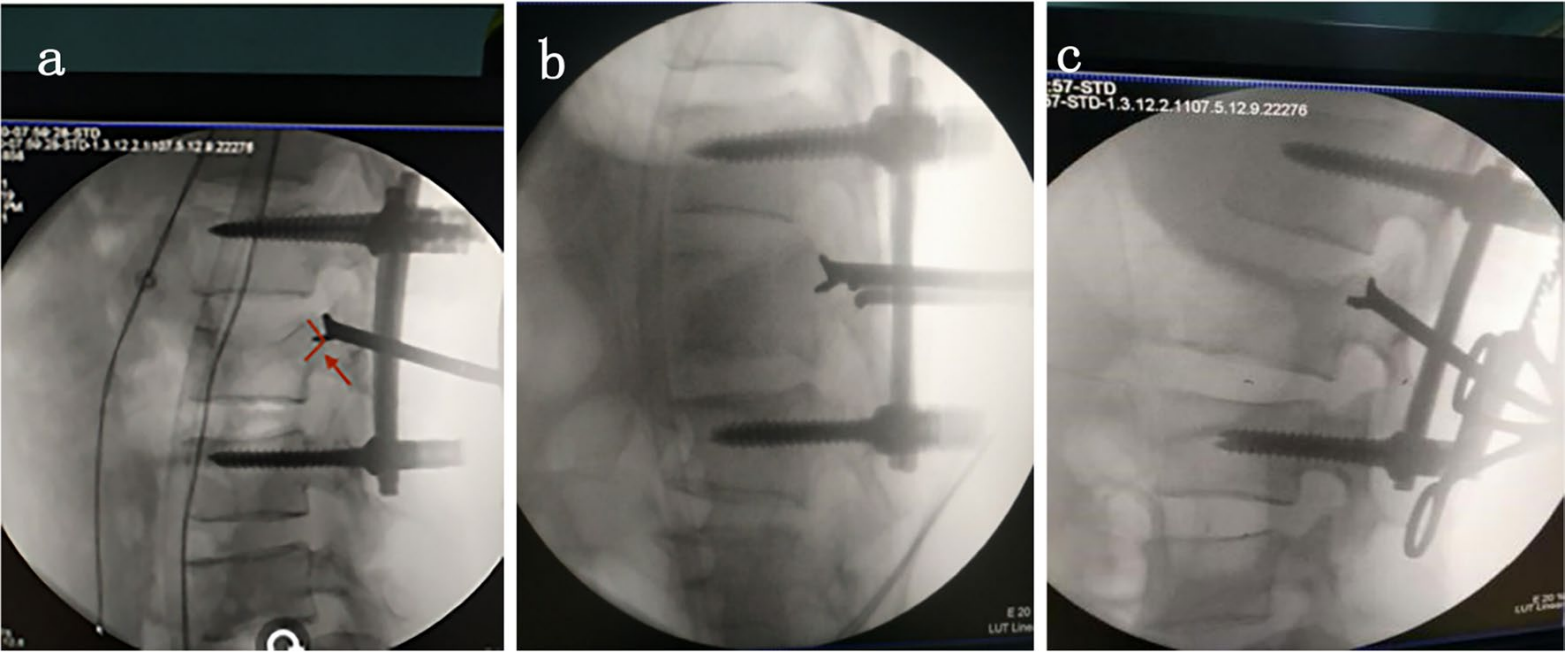
Reduction process for the Y-shaped fracture block under C-arm visualization during surgery. *Note*: The arrow in **a** shows the triangular fracture block, and the opening of the reduction device is about to anastomose the fracture block. **b** Shows the reset device anastomosing the fracture block. **c** Shows the reduction device pressing the fracture block into the vertebra

The same surgeon team treated both groups of patients, and the surgeon has been engaged in spine clinical work for twenty years. Conventional small posterior lamina space fenestration was adopted to expose the fracture pieces protruding from the spinal canal. Moreover, the proximal rod of the Y-shaped reduction was occluded vertically through the spinal canal, and external force was applied to the distal rod until the distal rod was perpendicular to the plane to press the fracture block into the vertebral body. If there was a bone defect in the injured vertebral body following reduction, the channel formed by the insertion of the lower lobe of the Y-shaped reductor was filled with allogeneic cancellous bone particles (Fig. 3).

### Data collection

The duration of the operation and intraoperative blood loss was recorded, and the ACRIV, Cobb angle, SSR, and ASIA nerve function were observed before the operation, immediately after the operation, and at least 3 months after the last follow-up. The ACRIV was calculated as the ratio between the average height of the anterior edge of the injured vertebrae and the sum of the heights of the adjacent upper and lower vertebrae. The SSR was measured on the transverse CT section according to the formula proposed by Hashimoto [15].

### Statistical analysis

SPSS version 23.0 (IBM) was adopted. The operation time, intraoperative bleeding, and the preoperative and postoperative SSR, ACRIV, and Cobb angle of the YRG and TRG were analyzed by *t*-test; to analyze the qualitative data on nerve function, the *Wilcoxon* test was used. *P* < 0.05 was regarded as critical.

## Results

All operations were accomplished, and no serious complications, including postoperative infection, neurovascular injury, loosening, or fracture of internal fixation, occurred. All patients were monitored for a mean of 14.2 months (range, 3–24 months). There were 20 patients in the YRG. And the operation time was 100–275 min, with an average of 190.6 min. The intraoperative blood loss was 60–400 ml, with an average of 241.5 ml. There were 20 patients in the TRG. And the operation time was 130–380 min, with an average of 236.6 min. The intraoperative blood loss was 150–900 ml, with an average of 357.20 ml. There were significant differences between the two groups in terms of operation time and blood loss, with those of the YRG being significantly lower than those of the TRG. In the YRG, the ACRIV was 58.9% preoperatively and recovered to 85.8% immediately after the operation and 84.9% at the last follow-up, with an average recovery of 26%. In the TRG, the average recovery was 54.7% prior to surgery and 85.4% at the most recent follow-up, for an average recovery of 30.7%. There was no significant distinction between the two groups. The average Cobb angle of the YRG was 16.1°, 8.8°, and 8.1° before, after, and at the last follow-up, respectively. In the TRG, the preoperative, postoperative, and final follow-up Cobb angles were 15.5°, 8.5° and 7.3°, respectively. And there was no significant distinction between the two groups. In the YRG, the mean SSR was 58.5% preoperatively and 23.4% postoperatively, and it recovered to 9.7% at the last follow-up. The SSRs of the TRG were 54.8%, 23.3% and 9.9%, respectively, and the SSR recovery was 44.9% at the last follow-up compared with the preoperative value. And there was no significant distinction between the two groups (Table 2). And there was no significant distinction in ASIA neurological function between the two groups (Table 3).

**Table 2:** Comparison of operation time, blood loss, and preoperative and postoperative SSR, Cobb angle, and ACRIV between the two groups

Groups	OT (min)	IB (ml)	SSR (%)			Cobb angle (°)			ACRIV (%)		
			Preop	Postop	At final	Preop	Postop	At final	Preop	Postop	At final
YRG	190.6 ± 53.8	241.50 ± 90.1	58.5 ± 10.4	23.4 ± 6.3	9.7 ± 2.1	16.1 ± 7.0	8.6 ± 3.1	8.1 ± 2.5	58.9 ± 12.9	85.8 ± 5.5	84.9 ± 4.8
TRG	236.6 ± 73.5	357.20 ± 179.1	54.8 ± 7.8	23.3 ± 5.6	9.9 ± 2.2	15.5 ± 6.8	8.5 ± 3.0	7.3 ± 2.2	54.7 ± 11.5	86.7 ± 3.0	85.4 ± 3.2
*P*	0.030	0.015	0.213	0.971	0.789	0.800	0.937	0.291	0.296	0.848	0.726

**Table 3:** Comparison of preoperative and postoperative ASIA classification between the two groups

Groups	Preop					Postop				
	A	B	C	D	E	A	B	C	D	E
YRG	4	2	5	8	1	1	1	2	3	13
TRG	3	2	7	6	2	1	2	1	3	12
*P*	0.904					0.901				

## Discussion

Fragments of a thoracolumbar burst fracture frequently protrude into the spinal canal and compress the spinal cord, resulting in cauda equina nerve and nerve root damage and corresponding neurological symptoms. Surgical intervention is usually recommended for the treatment of the burst fracture [20]. Various surgical procedures have been applied, including anterior, posterior, and combined approaches. Nonetheless, the anterior approach and the combined approach need long operation time, the trauma to patients is large and the risk is high, whereas the posterior approach is small and the risk is low. Accordingly, it is widely used in clinical practice [21, 22]. The posterior approach mainly includes minimally invasive procedures and open procedures. Percutaneous pedicle screw fixation has many advantages as a commonly adopted minimally invasive surgery for thoracolumbar fractures. Nonetheless, for severe unstable burst fractures, percutaneous pedicle screw internal fixation cannot fully decompress through the posterior longitudinal ligament [13, 23–25]. Hence, for direct reduction, open surgery is required.

**Fig. 4:**
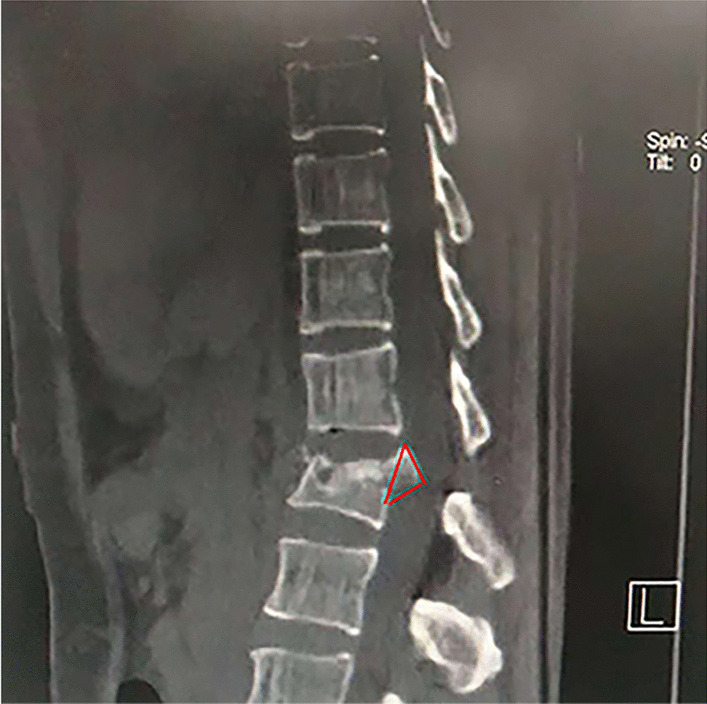
The shape of the fracture block is triangular, and the part protruding into the spinal canal has a Y-shaped opening

Posterior approach surgery requires the use of an appropriate fracture fragment reduction device for direct reduction [26]. Some reduction instruments used in the clinic can completely reduce the fracture block, but there are still drawbacks to their use, such as the requirement for laminectomy, which increases the damage to the lamina [27]. Posterior unilateral small fenestration of the lamina can reduce the damage to the posterior column of the vertebral body, and it becomes the main surgical method for the treatment of thoracolumbar burst fractures. Nonetheless, to reduce the fracture fragments in the small fenestration of lamina, some small and simple reducers are adopted to reduce the fracture fragments. Nonetheless, the free fracture fragments are not stable, and these reducers used during reduction cannot stably reset the fracture fragments, causing damage to the spinal cord or nerve root with minimal effort. Guerra et al. [28] indicated that most of the fracture fragments are in a “Y” shape, which is similar to our clinical observations. As we observed that the shape of the fracture fragments was more like a triangle (Fig. 4). Hence, according to the shape of the fracture fragments, the design of the Y-shaped fracture fragment reduction device meets the requirements for stable reduction of the fracture fragments under the condition of small fenestration and reduces the damage to the nerve and vertebral lamina. If the injured vertebra requires bone grafting, the channel formed by the lower slice of the Y-shaped in the vertebral body can be utilized.

**Fig. 5:**
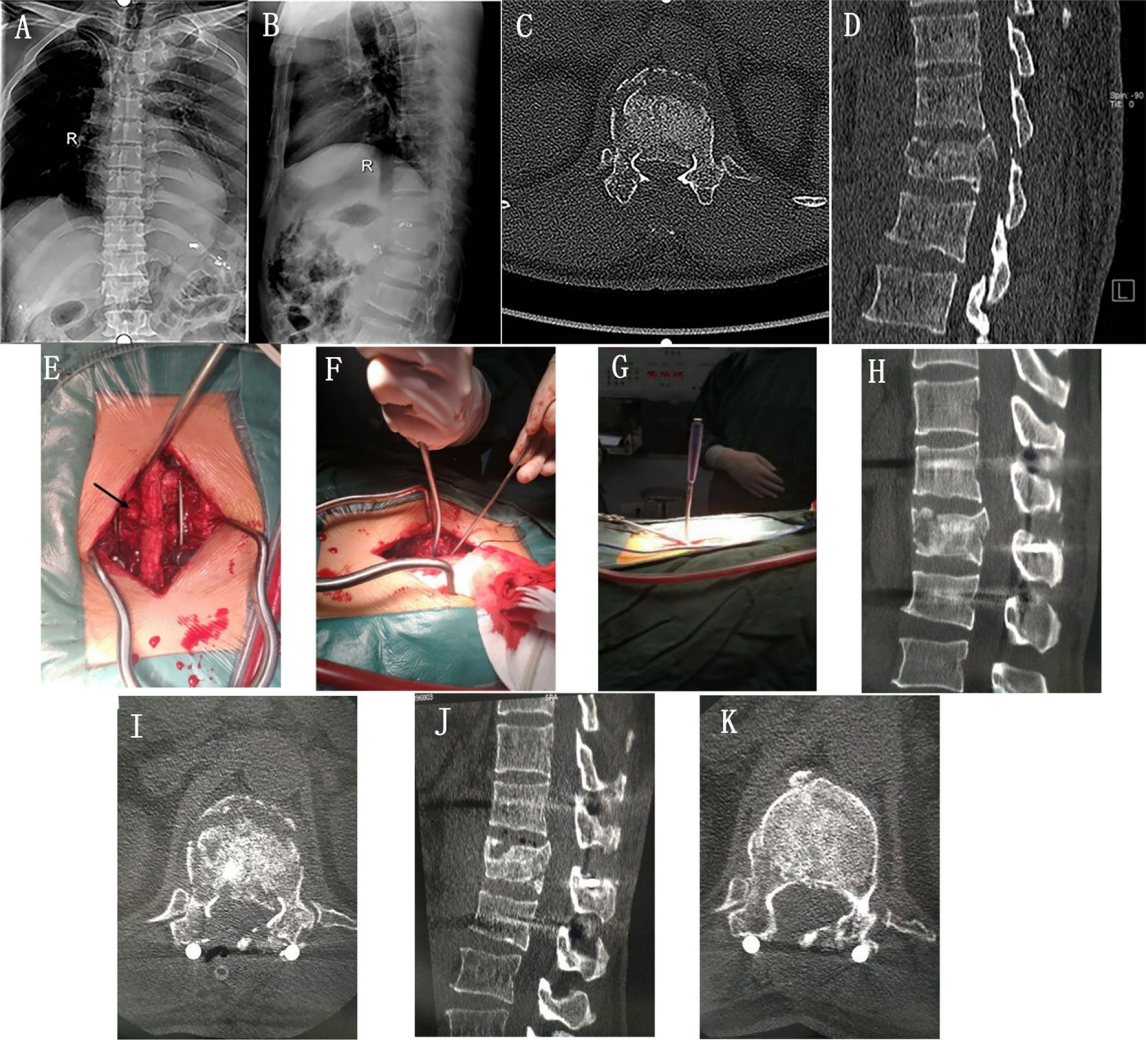
A 46-year-old male patient with a T_12_ burst fracture and spinal cord swelling injury (ASIA grade D). **A** and **B** are the anterior and lateral X-rays of the spine before surgery; **C** and **D** are preoperative CT coronal and sagittal images; **E**, **F**, and **G** refer to the use of Y-shaped fracture mass reduction device during surgery, with the arrow in **E** showing the size of the lamina space fenestration during surgery; **H** and **I** are the coronal and sagittal CT images immediately after surgery; **J** and **K** are the coronal and sagittal CT images from the last follow-up

In this study, there is no significant difference in SSR, ACRIV, ASIA neurological function, and Cobb angle between the YRG and the TRG. This implies that posterior small fenestration through intervertebral space is an effective method to treat thoracolumbar burst fracture. Nonetheless, in terms of operation time and intraoperative blood loss, there was a significant statistical distinction between the YRG and the TRG, and the YRG was lower than the TRG. This is due to the fact that the traditional reduction is mainly used to knock the reduction to drive the fracture block into the vertebral body due to the limitation of the force-bearing surface, the unstable way of percussion reduction is easy to cause a slip, which will increase the operation time. And repeated percussion will damage the blood vessels in the posterior wall of the vertebral body and increase the blood sinus in the cancellous bone in the vertebral body. Nonetheless, the front end of the Y-shaped fracture block reductor is designed according to the morphological characteristics of the overturned fracture block. Accordingly, when the reductor occludes the fracture block during the reduction process, it can stabilize the fracture block to prevent sliding during the reduction process and then press the fracture block into the vertebral body. This process can reduce the corresponding time compared to the conventional reset process. In addition, the Y-shaped fracture block reductor can completely press the fracture block into the vertebral body by occluding the fracture block. And the design of its front-end anastomosis slice can disperse the pressure on the posterior wall of the vertebral body, making its damage to the posterior wall of the vertebral body blood vessels and blood sinuses in the vertebral body relatively small. Hence, it can reduce the amount of intraoperative bleeding (Fig. 5).

After the procedure, patients in the two groups still had fracture fragments in their spinal canals, predominantly due to the elastic modulus of the screw rod system, changes in the postoperative posture, and increases in spinal activity, which attributed to the injured vertebra's fracture fragments to slightly protrude. Nonetheless, the fracture block is gradually absorbed during spinal canal remodeling. Studies have shown that [29, 30] fracture blocks generally begin to absorb gradually in 2–3 weeks, and the vertebral canal space occupation gradually decreases in 3–4 weeks. This process occurs within one year, so a fracture healing period of 3 months can effectively reflect the postoperative follow-up effect.

The anatomical features of the spine and the shape characteristics of the fracture block are the main design considerations for the Y-shaped fracture block reductor. In the process of reduction, it can not only meet the requirements of the small window opening but also stably occlude the fracture block to avoid displacement. Moreover, in terms of reduction mode, the Y-shaped reductor is employed to reduce the fracture block by pressing, which requires lower strength and higher reduction safety compared with knocking reduction. Furthermore, there are still some limitations in this study, including the number of cases included was small. As a result of the limited size of the spinal canal, the reductor is only suitable for thoracolumbar vertebrae below T_11_. To broaden the indications for the use of Y-type fracture block reductors, we will need to increase the number of cases, the sample size, and the reductor size in future.

## Conclusion

For patients with thoracolumbar burst fracture combined with severe spinal stenosis, the custom-made Y-shaped fracture block reduction device can effectively assist in posterior unilateral laminectomy to reduce the fracture block, as well as the reduction effect is positive. Besides, it can significantly reduce the duration of the operation and intraoperative blood loss.

## Data Availability

The datasets generated and analyzed during the current study are available from the corresponding author on reasonable request.
